# Study of the interactions of a novel monoclonal antibody, mAb059c, with the hPD-1 receptor

**DOI:** 10.1038/s41598-019-54231-w

**Published:** 2019-11-28

**Authors:** Jingxian Liu, Guiqun Wang, Liu Liu, Runjie Wu, Yi Wu, Cheng Fang, Xinhong Zhou, Jing Jiao, Ying Gu, He Zhou, Zhenhui Xie, Zhiwu Sun, Dakai Chen, Ken Dai, Dongxu Wang, Wei Tang, Teddy Tat Chi Yang

**Affiliations:** 1 Shanghai ChemPartner Co.Ltd, Shanghai, 201203 The People’s Republic of China; 2 Shanghai PharmaExplorer Co., Ltd, Shanghai, 201203 The People’s Republic of China

**Keywords:** Cancer, Immunology, Structural biology

## Abstract

Programmed cell death 1 (PD-1) monoclonal antibodies have been approved by regulatory agencies for the treatment of various types of cancer, and the mechanism involves the restoration of T cell functions. We report herein the X-ray crystal structure of a fully human monoclonal antibody mAb059c fragment antigen-binding (Fab) in complex with the PD-1 extracellular domain (ECD) at a resolution of 1.70 Å. Structural analysis indicates 1) an epitope, comprising fragments from the C’D, BC and FG loops of PD-1, contributes to mAb059c interaction, 2) an unique conformation of the C’D loop and a different orientation of R86 enabling the capture of PD-1 by the antibody complementarity determining region (CDR) and the formation of one salt-bridge contact – ASP101(HCDR3):ARG86(PD-1), and 3) the contact of FG with light chain (LC) CDR3 is maintained by a second salt-bridge and two backbone hydrogen bonds. Interface analysis reveals that N-glycosylation sites 49, 74 and 116 on PD-1 do not contact mAb059c; while N58 in the BC loop is recognized by mAb059c heavy chain CDR1 and CDR2. Mutation of N58 attenuated mAb059c binding to PD-1. These findings and the novel anti-PD-1 antibody will facilitate better understanding of the mechanisms of the molecular recognition of PD-1 receptor by anti-PD-1 mAb and, thereby, enable the development of new therapeutics with an expanded spectrum of efficacy for unmet medical needs.

## Introduction

Immune checkpoint inhibitors, especially anti-PD-1/PD-L1 therapeutic antibodies, have achieved great success in the area of oncology^1–3^. Subsequently, the PD-1 and PD-L1 pair was discovered based on their functions in T cell activity regulation, and monoclonal antibodies targeting the PD-1/PD-L1 interface have been designed to competitively block their interaction for therapeutic benefits^4,5^. Two anti-PD-1 antibodies, nivolumab (IgG4, Opdivo®) and pembrolizumab (IgG4, Keytruda®), were approved by the FDA in 2014^6^ and have demonstrated objectively effective responses in multiple cancers including melanoma, NSCLC and RCC^7–9^. Recent crystal structure studies of PD-1 and antibodies showed partially overlapped region but conformationally distinctive epitopes recognized by these two antibodies^10,11^. The dissociation constants of pembrolizumab and nivolumab on PD-1 are 27 pM and 1.45 nM, respectively, much lower than the dissociation constant of the PD1/PD-L1 interaction (8.2 µM)^11–14^, correlated with their complete blockade of PD1/PD-L1 binding.

There are four reported glycosylation sites, namely, N49, N58, N74, and N116, within the extracellular immunoglobulin variable (IgV) domain of PD-1^1,11,15^. Antibodies recognizing glycosylated PD-1 at these sites were reported to have a K_D_ ten times lower relative to deglycosylated PD-1. However, so far no structural evidence has validated the impact of N-glycan on PD-1 interaction with therapeutic antibodies^16^.

N58, which is on the BC loop of PD-1 and resides closest to the binding epitopes of pembrolizumab and nivolumab, was reported to be heavily glycosylated and most of the glycans consisted of two N-acetylglucosamines (GlcNac) and one fucose in the core position when PD-1 was expressed in both mammalian^11^ and insect cells^1^. Fucosylation has been associated with cancers^17^, and exhausted T cells in tumors carried highly core-fucosylated structures^15^. Overexpression of FUT8 and core fucosylation was observed in several cancers, such as lung and breast cancers^18,19^. Loss of core fucosylation caused PD-1 deprivation on the cellular surface and augmented T cell activation^15^. Physiologically, both TCR and PD-1 are glycoproteins, and core fucosylation could be utilized to regulate PD-1 expression by modulating TCR signaling strength^20^.

A recent crystal structure from an N-glycan study of PD-1 bound to nivolumab^11^ showed no direct contact of N58 glycan on PD-1 with nivolumab. In agreement, comparable K_D_ was observed in a binding study of glycosylated and deglycosylated PD-1 to nivolumab, indicating that the binding was glycosylation-independent. No reports on involvement of N-glycan on PD-1 binding to pembrolizumab are available. An antibody against PD-1, either expending the epitope areas of nivolumab and pembrolizumab, particularly the “hotspot”- FG loop^1^, or recognizing N-glycan, especially the N58-glycan in the BC loop will likely to facilitate a more comprehensive understanding of PD-1 and therapeutic antibody binding, and show differentiation to commercially available PD-1 antibodies.

In the present study, we report a 1.7 Å resolution crystal structure of PD-1 in complex with the Fab of a novel fully human PD-1 antibody, mAb059c. The epitope identified from the crystal complex structure is different from those of nivolumab and pembrolizumab. Additionally, the N58 glycan on PD-1 is shown to interact with mAb059c, with a binding affinity ~50-fold higher than that of deglycosylated PD-1 with mAb059c.

## Results

### The C’D and FG loops of PD-1 dominate the binding to mAb059c

**Figure 1:**
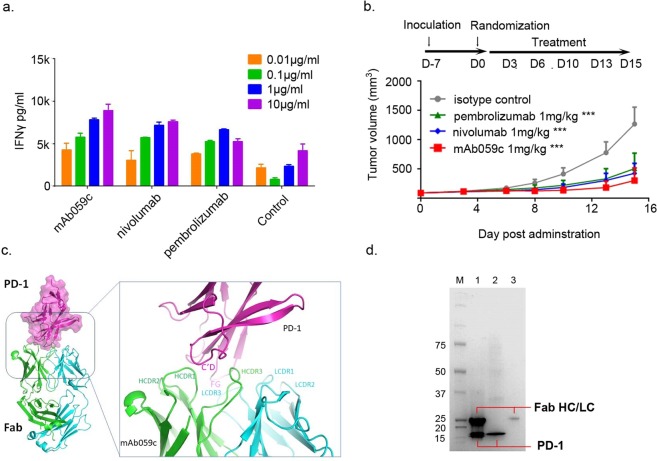
Biologically relevant assembly of the PD-1-mAb059c complex structure. (**a)** Mixed Lymphocyte Reaction Assay. IFN-γ release is measured in the presence of different doses (10, 1, 0.1, 0.01 µg/ml) of mAb059c, nivolumab, pembrolizumab and control; Similar dose-dependent enhancement of the IFN-γ secretion by mAb059c, nivolumab, pembrolizumab references are observed with multiple DC and T-cell donor pairs. (**b)** *In vivo* efficacy study using the MC38 model in hPD-1 knock-in mice at a dose of 1 mg/kg. ***P < 0.001 vs IgG via Two-way ANOVA with Bonferroni multiple comparison test. Similar results are observed at 10 mg/kg dose (Tumor growth curves with all groups are shown in Supplementary Fig. S1); (**c)** The complex structure of PD-1-mAb059c is displayed as a cartoon representation. The surface of PD-1 is shown in purple. The heavy chain and light chain of mAb059c are shown in green and cyan, respectively. C’D/FG loops of PD-1 and the HCDR and LCDR loops of mAb059c are labeled in purple, green and cyan, respectively; (**d)** The mAb059c Fab-PD-1 association validated by SDS-PAGE gel. Lane 1, crystal harvested from 3 droplets (roughly 15~30 µg) and dissolved in well solution after washing 2 times; Lane 2, 2 µg of PD-1 alone; Lane 3, 1 µg of mAb059c Fab alone; the rest of the blank area in the gel image was cropped (the full-length gel is presented in Supplementary Fig. S2).

Fully human anti-PD-1 antibody mAb059c was generated with the hybridoma approach by immunizing 6-8-week-old Harbour H2L2 transgenic mice (Supplementary Section). Purified mAb059c bound to human and cynomogous PD-1 proteins with an affinity of 36 pM and 45 pM by ELISA, respectively. mAb059c blocked PD-1 and PD-L1 interaction with an IC50 of 1.6 nM (data not shown). A mixed lymphocyte reaction (MLR) assay showed mAb059c induced comparable enhancement of interferon-γ production and T cell activation to nivolumab and pembrolizumab references (Fig. 1a). Additionally, an *in vivo* efficacy study using the MC-38 model in human PD-1 (hPD-1) knock-in mice showed that mAb059c was as efficacious as pembrolizumab and nivolumab references at a dose of 1 mg/kg (Fig. 1a,b) and 10 mg/kg (Supplementary Fig. S1). To gain further insight into the molecular mechanism of immune checkpoint blockade by mAb059c, a co-crystal of mAb059c Fab and PD-1 ECD (extracellular domain) complex was solved at 1.7 Å resolution. The collection and refinement statistics are shown in Table 1. The region N33-R148 of PD-1 was built by molecular replacement. The fragments in both ends were not resolved due to the absence of electron densities in these regions. One PD-1 and one mAb059c molecule were found in one asymmetric unit. The overall complex structure and molecular recognition in the PD-1 loop regions are illustrated in Fig. 1c. The interface area was calculated as 757 Å^2^ (538 Å^2^ in HC and 219 Å^2^ LC), and the heavy chain of mAb059c dominated in the binding with PD-1. In summary, the epitope is composed of fragments from the BC (residues 61–64), C’D (residues 83–86) and FG (residues 126–134) loops, which contact heavy chain CDR (HCDR) 2, HCDR3 and light chain CDR3 (LCDR3) of mAb059c, respectively (Fig. 1c). The interaction of the refolded PD-1 extracellular domain with mAb059c Fab was also verified by testing the complex crystals in SDS-PAGE, as shown in Fig. 1d.

**Table 1:** Data collection and refinement statistics.

	PD-1-mAb059c
**Data collection**	
Space group	P2_1_2_1_2_1_
Wavelength	0.97916
**Cell dimensions**	
*a*, *b*, *c* (Å)	39.95, 102.59, 137.72
α, β, γ (°)	90.00, 90.00,90.00
Resolution (Å)	41.9–1.70 (1.70–1.73)^a^
*R*_sym_ or *R*_merge_	0.166(1.061)
CC_half	0.994(0.796)
*I*/σ*I*	40.0(5.2)
Completeness (%)	98.21% (84.71%)
Redundancy	10.0 (7.0)
**Refinement**	
Resolution (Å)	41.9–1.70
No. reflections	62243
*R*_work_/*R*_free_	0.171/0.214
No. atoms	4684
Protein	4176
Ligand/ion	16
Water	492
B-factors (all atom)	18.7
Protein	17.6
Ligand/ion	28.5
Water	27.6
**R.m.s. deviations**	
Bond lengths (Å)	0.0037
Bond angles (°)	1.24
**Ramachandran plot**	
Favored (%)	98.3
Allowed (%)	1.7
Outliers (%)	0

(^a^highest resolution shell is shown in parenthesis).

**Figure 2:**
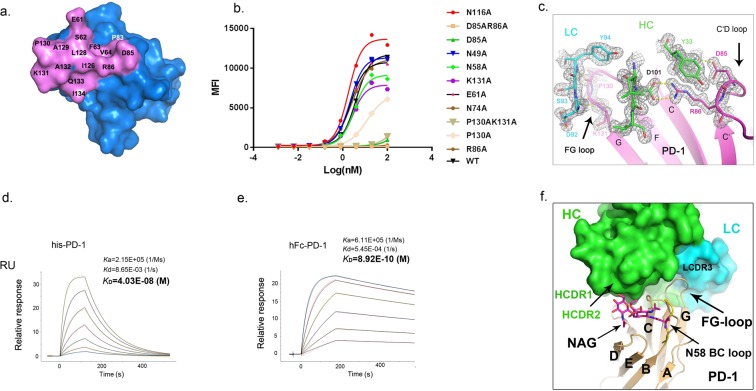
The binding interaction between mAb059c and PD-1. (**a)** The binding footprint on PD-1 recognized by mAb059c. The PD-1 surface is shown in blue, and the mAb059c epitope region is shown in violet. Individual residues contributing to the binding are labeled. The footprint is selected by a distance of 5 Å around mAb059c; (**b**) The electron density map 2Fo-Fc is generated by the FFT program in CCP4 and displayed by Pymol. Residues from the FG loop (130–131) and C’D loop (85–86) of PD-1, HCDR3 (100–102), LCDR1 (92–94) and Y33 of mAb059c are shown with stick representation surrounded by an electron density map; (**c,d**) K_D_ measurement of deglycosylated his-PD-1 (**c**) or glycosylated hPD-1 (**d**) by BIAcore 8 K; (**e**) The fitting curves of different mutants on critical residues. Y axis – mean fluorescence intensity. X axis – Log(nM). (**f**) Superimposition of PD-1 of 5WT9 and the PD-1 of 6K0Y. For simplicity, the structure of 5WT9 is not shown except for the BC loop fragment (residues 56–59, yellow) of 5WT9 (in cartoon representation). The N-glycan at N58 GlcNac(FUC)-GlcNac is shown with magenta stick representation.

The conformational epitope is composed of a few residues exposed on the PD-1 protein surface, as illustrated in Fig. 2a. Several residues are involved in hydrogen bond and salt-bridge interactions (Supplementary Table S1). Among these, R86 from the PD-1 C’D loop forms a salt-bridge linkage with D101 from HCDR3 of mAb059c, while the Y33 from HCDR1 stacks in parallel with R86 in PD-1, which serves to orient the arginine side-chain^21^ and aids the bridge interaction. The neighboring residue D85 in PD-1 also connects S32 in HCDR1 via hydrogen bond. Mutations of these residues (R86A, D85A and D85AR86A) abolished the PD-1 and mAb059c interaction as shown in a cell-based binding experiment (Fig. 2b). Similar to the nivolumab crystal structure, the FG loop of PD-1 in mAb059c complex structure is also involved in molecular recognition by mAb059c. The loop fragment A129-P130-K131-A132 behaves in a bended manner, aligning with D92-S93-Y94 from mAb059c via one salt-bridge (K131-D92) and two main-chain hydrogen bonds, as shown in Fig. 2c. In agreement, the mutations on residues P130 and K131 resulted in attenuated binding affinities, as evidenced by cell-based binding assay, whereas double mutations of these two residues completely abolished the binding (Fig. 2b). One additional hydrogen bond was found between the main chain of A132 (PD-1) and side-chain of D92 (mAb059c). The BC loop (residues 61–64) was observed to connect mAb059c HCDR2 via E61 hydrogen bond and van der Waals interactions, but mutation of E61 didn’t affect the overall recognition, indicating the minimal role of this residue in binding as shown in Fig. 2b.

### N58 glycosylation-dependent binding to mAb059c

To explore the possible involvement of glycosylation in PD-1 recognition, we measured the mAb059c binding affinity for both glycosylated and deglycosylated PD-1 by SPR. Surprisingly, the binding affinity for glycosylated hPD-1 protein (8.92 × 10^−10^ M) is nearly 50-fold higher than that for deglycosylated hPD-1 (4.03 × 10^−08^ M), as illustrated in Fig. 2d,e. Structural analysis reveals that three glycosylation sites, N49, N74, and N116, are distant from the interface, while the glycosylation site N58 on PD-1 is very close to the binding interface, similar to the observation described in the structural study of nivolumab^11^. It was reported that the N-linked glycosylation modification at N58 of PD-1 consisted of two N-acetylglucosamines (GlcNac) and one fucose, when expressed and purified from mammalian cells^11^. MS analysis of PD-1 expressed in 293 F cells confirmed that the (GlcNac)2-Fuc structure was observed in most types of glycans attached to the _57_SNTSESF_63_ peptide of PD-1 (Supplementary Table S2). A previous structural study showed no contact between the glycosylated residue (N58) and the CDR residues on nivolumab^11^. Further affinity measurement of both deglycosylated and glycosylated PD-1 with nivolumab did not show much difference. By contrast, in the present study, the binding affinity of mAb059c to glycosylated PD-1 is significantly higher than to deglycosylated PD-1. Detailed structural information is revealed by comparing structures of glycosylated PD-1 bound to mAb059c against the glycosylated PD-1 bound to nivolumab (5WT9) (Fig. 2f). The calculated overall RMSD value is 0.649 (calculated by align function in Pymol), and all of the residues align well in two structures, except for the FG loop region (residues 128–133) and the N58 backbone region. The N-glycan on the N58 site folds into the groove between the BC loop, DE loop, and C’D loop near the C’ strand, which is involved in protein folding, function of PD-1 and binding with mAbs^17^. The fucose branching at the first N’acetylglucosamine on PD-1 remains close to heavy chain CDR1 and CDR2 of mAb059c. In particular, R30 from mAb059c HCDR1 is in close proximity to PD-1, while the long side-chain of R30 displays a bended rotamer conformation in the structure. Furthermore, the cell-based binding experiment showed that mutations of N49, N74 and N116 do not affected mAb059c binding; however, mutation of the N58 of PD-1 resulted in an attenuated interaction between PD-1 and mAb059c (Fig. 2b). These data suggest that N58 glycosylation contributes to mAb059c binding to PD-1.

### mAb059c completely competes with pembrolizumab and PD-L1 but partially competes with nivolumab binding to PD-1

**Figure 3:**
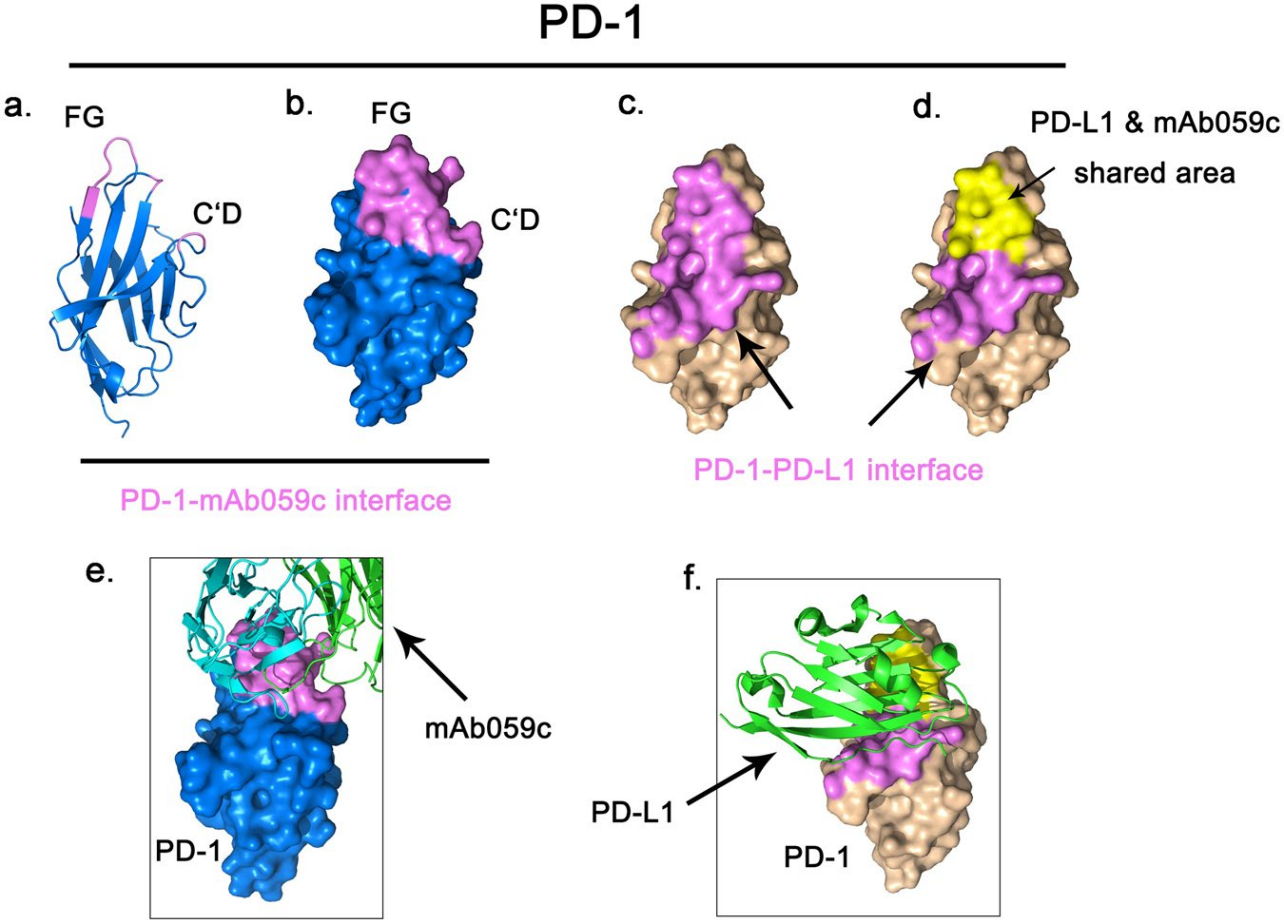
mAb059c shared with PD-L1 in their binding area. (**a)** A cartoon representation of PD-1; (**b)** The mAb059c binding footprint is indicated in violet; (**c**) The PD-1 (4ZQK) surface is shown in wheat, and the PD-L1 binding footprint is indicated in violet; (**d)**. The shared area in both the mAb059c and PD-L1 binding footprint is indicated in yellow; (**e)**. Fig. b overlaid by mAb059c (in stick representation); (**f)**. Fig. d overlaid by PD-L1.

**Figure 4:**
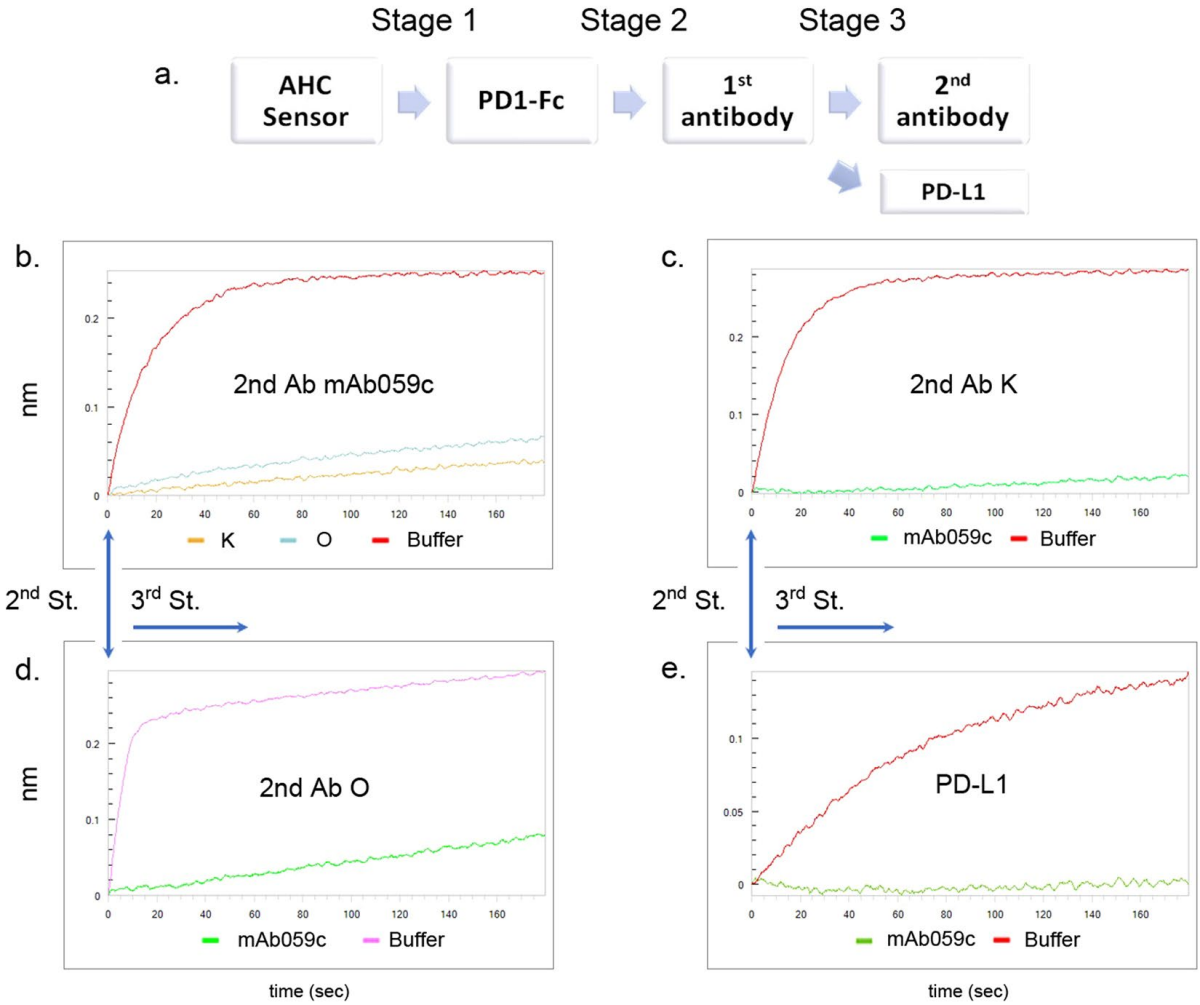
BLI competition assay. (**a)**. Flow-chart of the BLI competition assay using Octet. b-e are the curves of stage 3. (**b**) mAb059c is injected as a 2^nd^ antibody; (**c**). Pembrolizumab is injected as a 2^nd^ antibody; (**d**). Nivolumab is injected as a 2^nd^ antibody; (**e**). PD-L1 is injected after mAb059c is immobilized on the membrane. O and K stand for Nivolumab and Pembrolizumab respectively.

The competitive relationship of mAb059c with PD-L1 was evaluated to better understand the mechanism of action of PD-L1 blockade by mAb059c. As shown in Fig. 3a,b, mAb059c interacts with the loop regions of PD-1 (C’D, FG and BC loops), while the PD-1 binding interface with PD-L1 is the front face of the β-sheet shown in Fig. 3c according to previously published PD-1/PD-L1 structure (4ZQK)^22^. Intriguingly, superimposition of the PD-1/PD-L1 structure and PD-1/mAb059c structure reveals that the overlapping region of these two interfaces is mainly composed of loop regions of strands of FGC indicated by yellow color in Fig. 3d. This shared area is recognized by both HC and LC of mAb059c (Fig. 3e) and PD-L1 (Fig. 3f) respectively. The binding competition between pembrolizumab/nivolumab/PD-L1 and mAb059c was further evaluated by Bio-layer Interferometry (BLI) experiment and the flow of injection is demonstrated in Fig. 4a. A notable difference between nivolumab and pembrolizumab was observed: nivolumab partially while pembrolizumab completely blocked the binding of mAb059c to PD-1, in both the injection sequence of mAb059c-K/O and K/O-mAb059c during Stages 2–3 (Fig. 4b–d). The result of PD-L1 competition assay showed that mAb059c completely blocked the binding of PD-L1 to PD-1 (Fig. 4e).

**Figure 5:**
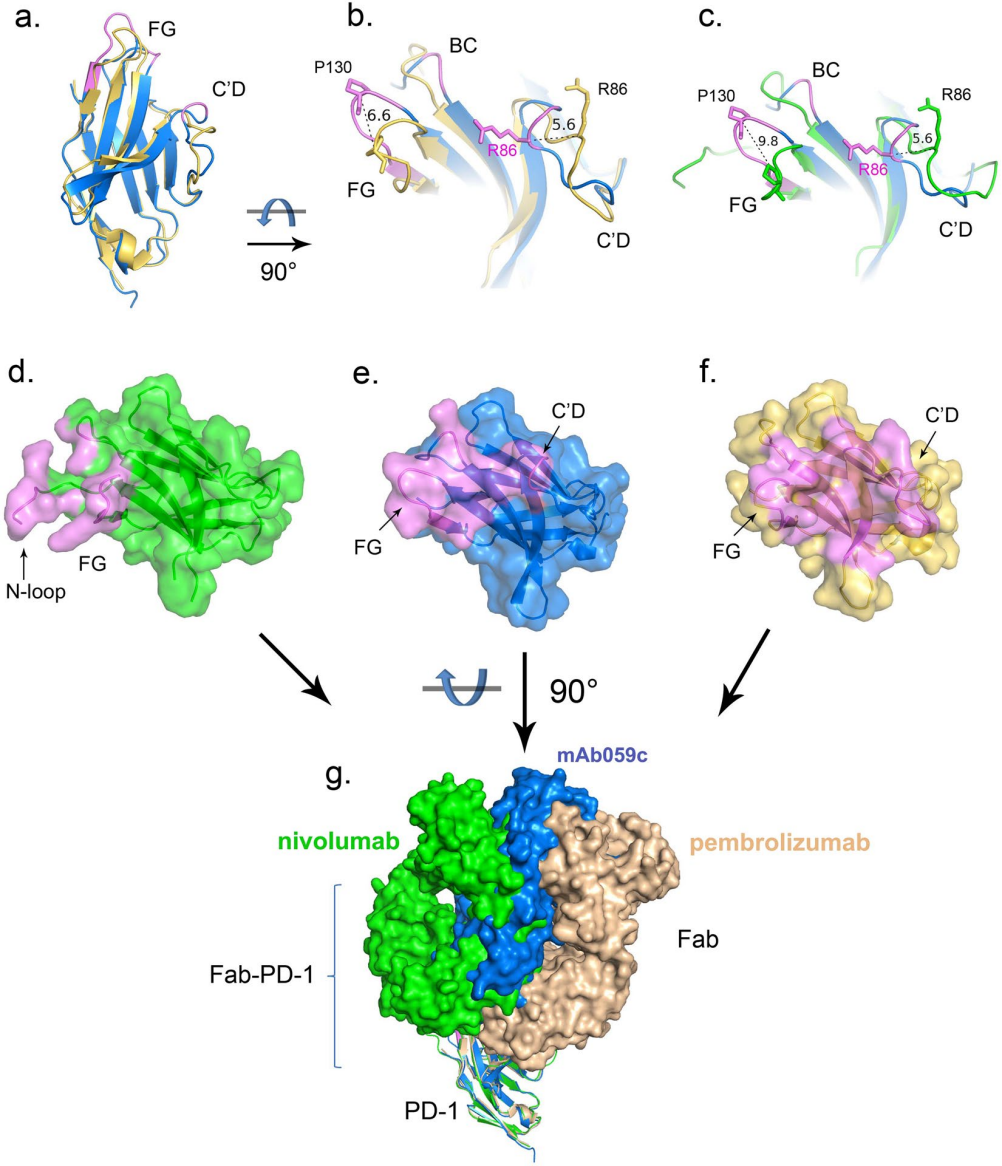
Epitope comparison PD-1 structures of mAb059c-PD-1, pembrolizumab-PD-1 and nivolumab-PD-1 are superimposed in Pymol. Epitopes are shown in purple color. (**a**) PD-1 of mAb059c-PD-1 (blue) and pembrolizumab-PD-1 (wheat) are superimposed in cartoon representation; (**b**). Zoom-in view of fig. a; (**c**). Zoom-in view of the PD-1 structures of mAb059c-PD-1 (blue) and nivolumab-PD-1 (green); distances between R86 (C’D loop) residues and distances between P130 (FG loop) residues from individual structures are indicated with black dashed lines; (**d**). Surface of PD-1 from nivolumab is shown in green; **e**. Surface of PD-1 from mAb059c is shown in blue; (**f**). Surface of PD-1 from mAb059c is shown in wheat; g. Relative postures of the Fabs (with surface representation) are shown in this figure. From left to right in sequence - Green: nivolumab, Blue: mAb059c, Wheat: pembrolizumab. PD-1s superimposed are shown with cartoon representation.

By comparing mAb059c crystal structure with the nivolumab or pembrolizumab structures, significant conformational changes in both the FG and C’D loops are observed (Fig. 5a). In particular, the FG loop shifts ~9.8 Å away from the one observed in the nivolumab structure, and the backbone of R86 in the C’D loop shifts ~5.6 Å away relative to the nivolumab and pembrolizumab structures (Fig. 5b,c). Intriguingly, mAb059c structure shows a twisted C’D loop fragment 85–86 and a totally different orientation of the R86 side-chain, which participates in the formation of hydrogen bonds. Comparison of a previous structural study^11^ and the current structural analysis of PD-1 - mAb059c complex demonstrates that the epitope covered by nivolumab is mainly from the N-loop of PD-1, while mAb059c and pembrolizumab both recognizes the FG and C’D loops (Fig. 5d–f); thus, the binding interfaces of mAb59c and pembrolizumab are different from those of nivolumab but similar to each other. Intriguingly, antibodies show different “sitting” angles after superimposition of the 5GGS, 5GGR and 6K0Y PD-1 structures. mAb059c is sandwiched by pembrolizumab and nivolumab, and each orientation is influenced by its different recognition focus (Fig. 5g).

## Discussion

The ultimate goal of immune checkpoint blockade is to achieve desirable therapeutic efficacy via the blockade of PD-1/PD-L1 interaction. mAb059c is of the IgG4 subclass. IgG4 lacks antibody-dependent cell-mediated cytotoxicity (ADCC) function and only weakly induces complement and cell activation due to low affinity for the C1q and some Fc receptors^23^. The comparison of PD-1 recognition by different antibodies is critical to understand the nature of steric competition in the blockade of PD-L1 binding to PD-1. Both mAb059c and pembrolizumab recognize C’D via the HCDR3 salt-bridge interaction. In mAb059c, a unique orientation of the R86 side-chain is observed, accompanied by a twisted backbone trace in the D85-R86 fragment, while in a pembrolizumab study, D85 is found to form a salt-bridge with its counterpart^21^. Similarly, both mAb059c- and nivolumab-PD-1 structures show the molecular recognition of the PD-1 FG loop via the antibodies’ CDR regions. Particularly, LCDR3 of mAb059c pairs with the FG loop of PD-1, which shows a bended conformation, whereas in the nivolumab study, the FG loop turn region interacts via hydrogen bonds and fits into the binding groove created by the heavy chain and light chain of nivolumab. Additionally, the N-loop of PD-1 was identified to be the dominant component interacting with nivolumab^21^. The unique binding pattern in each PD-1-antibody pair potentially could lead to different mechanisms of action for the blockade of PD-L1 interaction with PD-1, and therefore different efficacies.

PD-1 and PD-L1 interact through the conserved front (A’GFCC’) β-sheet and side of their Ig variable (IgV) domains, similar to the IgV domains of antibodies and T cell receptors^12^. Specifically, the C strand, CC’ loop, C” strand, C’ strand, CD loop, FG loop and G strand from PD-1 interacts with individual strands from PD-L1. Among these, pembrolizumab bound the C strand (N66), C” strand (T76), C’ strand (K78) and FG loop (K131, A132)—a total of four out of seven strands/loops on the PD-1/PD-L1 interface. D85 in the C’D loop of PD-1, which forms a salt-bridge with pembrolizumab, also contributes to the blockade of PD-L1 binding to PD-1. Similarly, the blockade of PD-1 and PD-L1 binding by nivolumab is attributed to blocking two regions on PD-1 surface, namely, the FG loop and N-loop. The FG loop (L128, P130, K131), bound by nivolumab, is also shared by the PD-1/PD-L1 interface, whereas the N-loop identified as a key interaction in the nivolumab study, is not shared by the PD1/PD-L1 interface. In the present study, several hydrogen bonds or salt-bridges were identified through structural analysis. The P130-K131-A132 fragment in the FG loop forms hydrogen bonds with mAb059c and completely blocks the PD-1 interaction with PD-L1. However, other residues, E61, D85, and R86 of PD-1, which are not in the regions on the PD-1/PD-L1 interface, contribute to overall binding by mAb059c to PD-1 and prevent PD-L1 from binding via enhanced blockade effect on the FG loop.

A competition experiment demonstrated that mAb059c binding to PD-1 was partially blocked by nivolumab and completely blocked by pembrolizumab. Both nivolumab and pembrolizumab binds flexible loops of PD-1 but with different emphasis: The N-loop by nivolumab and the C’D loop by pembrolizumab. These two regions on the PD-1 surface span quite a long distance; therefore, almost no overlapping of the epitope interfaces is detected in structural analysis of these two antibodies^11^. The orientations of nivolumab and pembrolizumab, after superimposition of their binding partner PD-1, differ markedly. Nivolumab and pembrolizumab are the two therapeutic antibodies most frequently used in clinic with similar clinical efficiencies in some cancer type and different efficacy in some other cancer type^7,24–26^, indicating binding epitope may contribute to the differences in efficacy. Interestingly, the combined involvement of the C’D and FG loops in the binding of PD-1 by mAb059c, as confirmed by structural analysis and cell-based binding study, leads to its binding site “sandwiched” by those of nivolumab and pembrolizumab. Therefore, the BLI result could be explained by the different binding focus in all loops, including the N-loop, BC, C’D and FG. Nivolumab and mAb059c both recognize the FG loop, but nivolumab mainly binds the N-loop of PD-1^11^. Pembrolizumab and mAb059c both bind the C’D loop, which is main focus in both situations.

The unexpectedly high binding affinity (subnanomolar) of glycosylated PD-1 by mAb059c in SPR measurement prompted further analysis of the role of glycosylation in affinity enhancement. Glycans play an important role in immune surveillance^27^. Glycans also alter protein conformation and structure, thus modulating the functional activity of a protein^28^. To evaluate the effect of glycosylation on PD-1 binding by mAb059c, the N58 glycosylated PD-1 complex structure^11^ is superimposed with deglycosylated one. N58 GlcNac(FUC)-GlcNac fragment is shown to be in close proximity to the binding interface between PD-1 and mAb059c. The long side-chain of R30 from HCDR1 of mAb059c, which is in a bended rotamer conformation in the glycan-free structure, possibly contributes to the affinity enhancement via N58 glycan hydrogen bond connection. In addition, mutations on the remaining three glycosylation sites do not show alteration of signals relative to the WT control, indicating N58 glycosylation might be the sole contributor affecting glycosylated PD-1 binding by mAb059c. In addition, although the glycan types are complicated, most of them are core fucosylated according to the MS results (Supplementary Table S2) and the structures of different glycans^29^. Modulation of glycosylation facilitates escape from immune surveillance^15,18,19^. However, the involvement of possible core fucoses in binding could be an advantage to facilitate interaction of different antibodies with PD-1 with enhanced binding affinity, and may lead to better clinical results^30^.

The desired blockade effect could be achieved by designing more direct interactions to compete the binding of PD-L1 on the front β-sheet of PD-1, as depicted in the analysis of pembrolizumab, or by increasing the blocking effect of the “hotspot” FG loop through enhancing the binding affinity via the introduction of more hydrogen bonds in different regions such as the C’D loop. *In silico* tools such as quantum mechanics are good approaches to delineate the profile of residue-residue interactions of PD-1/PD-L1 from the binding energy point of view, thus facilitating the design of better therapeutic antibodies targeting ideal conformational epitopes^31,32^.

In summary, a new anti-PD-1 antibody, mAb059c, with subnanomolar binding affinity that targets a new epitope including the C’D loop, FG loop and BC loop is described in this study. The involvement of the N58 glycosylation in PD-1 recognition by mAb059c, confirmed by an ~50-fold K_D_ enhancement, structural analysis and cell based binding analyses, extend the epitope further to the BC loop, making it a useful tool to better understand the biology and modes to block PD-1/PD-L1 binding. This newly discovered epitope broadens the spectrum of the “hotspot” loops of PD-1 that are targetable by therapeutic antibodies and may potentially diversify the strategies for the development of biologic drugs for immune checkpoint blockade.

## Methods

### Protein expression and purification

The constructed PD-1 (25–167) sequence was ligated into the pET-41a vector, transfected into *E. coli* BL21 and cultured. The solubilized hPD-1 protein was refolded by fast dilution in buffer consisting of 1 M arginine-HCl, 100 mM Tris-HCl (pH 8.0), 2 mM NaEDTA, 0.5 mM reduced glutathione, and 0.05 mM oxidized glutathione at 4 °C overnight. Purified PD-1 was concentrated and further exchanged with PBS to a final concentration of 7.4 mg/ml.

Vector containing hPD1(25–167)-TEV-Fc was transfected in HEK293 cells and cultured for 7 days before harvest. The fusion protein was purified by a MabSelect SuRe Protein A column (GE, China) followed by HPLC-SEC purification.

Vector encoding Fab of mAb059c (hIgG4/hKappa) was transfected in 293 F cells and cultured for 7 days before harvest. The Fab was purified by KappaSelect affinity chromatography, concentrated to 3 mg/ml and frozen in liquid nitrogen for future usage.

PD-1 and mAb-059C was mixed at 1:2 (v/v) for crystallization at room temperature.

### *In vitro* mixed-lymphcyte reaction (MLR)

Purified CD4^+^ T cells were cocultured with allogeneic monocyte-derived DCs in the presence of a titration of different anti-PD-1 antibodies or isotype control antibody. Supernatants were collected and measured for IFNγ by ELISA.

### *In vivo* efficacy study using the MC38 model in hPD-1 knock-in mice

Animal studies were performed in specific pathogen-free (SPF) animal facility according to protocols approved by ChemPartner Institutional Animal Care and Use Committee (IACUC) following Assessment and Accreditation of Laboratory Animal Care (AAALAC) guidelines. Seventy male human PD-1 knock-in C57BL/6 mice (21~23 g) were purchased from Nanjing Galaxy Biopharmaceutical Co., Ltd., Nanjing, China. Animals were held for 6 days for acclimation prior to inoculation of murine colon carcinoma MC-38 cells (acquired from National Cancer Institute, Frederick, MD, USA) at 1 × 10^6^ cells/mouse in 0.1 ml PBS subcutaneously into the right flank. Animals with tumors 60 mm^3^ ~ 110 mm^3^ in size were selected 7 days after tumor inoculation and randomly divided into treatment groups based on their tumor sizes. Antibody treatments, in 0.1 ml PBS by i.p. injection twice weekly for 2 weeks, started on the day of randomization (defined as D0). Mice were treated in groups described in Table S3. Tumors were measured three times per week using a digital caliper, and tumor volumes were calculated: Tumor volume = (length × width^2^)/2. Figures of tumor growth curves were plotted using GraphPad Prism 5.

### Statistical analysis

Statistical analysis was performed using GraphPad Prism 5. Two-way ANOVA with Bonferroni multiple comparison test was used to compare tumor growth and body weight changes (data not shown) over time between different treatment groups. The difference was considered statistically significant if P < 0.05.

### Analysis of the binding of PD-1 mutants to mAb059c by flow cytometry

Full length human PD-1 was cloned into the pIRES-puro vector. Four reported glycosylation sites, 49, 58, 74 and 116, were mutated to Ala by site-directed mutagenesis. Key interaction sites E61, D85, R86, D85&R86, P130, K131, and P130&K131 were also selected and mutated to alanine by site-directed mutagenesis. The plasmids were transfected into 293 F cells to express different PD-1 mutants.

Cells were incubated with 100 µl of diluted antibody solution (3 µg/ml) at 4 °C for 1 h followed by washing and incubating with secondary Ab (Alexa488 anti-human IgG, 2 µg/ml) at 4°C for 1 h. The cells were analyzed with a BD FACS Aria II (BD Biosciences, San Jose, CA, USA).

### Crystallization and data collection

Co-crystallization of glycosylated PD-1 or PD-1 digested with PNGase F enzyme with mAb059c was attempted, but not successful. A crystal of refolded PD-1 was observed in ProComplex C08 conditions after incubation for three days. Further condition optimization generated crystals with a long shape in the condition consisting of 0.2 M NaCl, 0.1 M Tris pH 8, and 14% PEG 4 K. Crystals were incubated in wells with a solution containing 20% glycerol and then flash-frozen in liquid nitrogen. Data were collected on BL17U at SSRF with a helical approach. Five data sets were processed and integrated by HKL2000. The phaser at CCP4 was used for molecular replacement, and the searching model of mAb059c was generated by Bioluminate with the Schrodinger package using the homolog modeling method. The searching model of PD-1 was from 5GGS. Model building and refinement were performed with COOT and Refmac in CCP4. Structure factor files and atomic coordinate files were deposited in PDB with code 6K0Y. Data collection and refinement statistics are summarized in Table 1. Structure demonstrations were performed by PYMOL software (http://www.pymol.org).

### K_D_ determination by SPR

SPR affinity measurement of both glycosylated and refolded PD-1 with mAb059c was performed on a BIAcore 8 K (GE Healthcare, China) with a CM5 chip (anti-his and anti-hFc, GE Healthcare). Capture of PD-1 was followed by two-fold dilution series of mAb059c from 200 nM to 3.125 nM (50 nM to 1.5 nM for PD1-hFc). Each analyte was run with a contact time of 120 s and dissociation time of 400 s. The binding kinetics were all analyzed with the software BIAevaluation Version 4.1 using a 1:1 multicycle kinetic model.

### Binding competition assay

Binding competition between mAb059c and pembrolizumab/nivolumab was evaluated by a label-free biolayer interferometry assay on an Octet Red 384 (Pall ForteBio, USA). All experiments were performed at 25 °C in PBS buffer with 0.005% Tween-20 (PBST). PD1-hFc was loaded onto AHC sensors at a concentration of 2 µg/ml for 300 s in Stage 1, and then the 1^st^ antibody (100 nM) was loaded onto the biosensor for 600 s to obtain saturation in Stage 2. The second antibody was loaded on the biosensor at a concentration of 100 nM for another 600 s in Stage 3. The above experiments were repeated in the reverse order of antibody loading in Stages 2 and 3. PBST buffer was used as the negative control. The competition between mAb059c and PD-L1 was evaluated in similar way except that mAb059c and PD-L1 were loaded onto the biosensor sequentially in Stages 2 and 3. All sensors were regenerated using 10 mM glycine-HCl buffer (pH 1.7, GE Healthcare), and recharging was done in 10 mM NiCl2 buffer. The real-time binding response was measured during the course of the experiment. The binding responses of the two commercial antibodies and mAb059c to PD-1 were compared with the response from the negative control (PBST), and competitive/noncompetitive behavior was determined.

### Data deposition

The atomic coordinate file and structure factor were deposited with code ID 6K0Y. Structures of nivolumab Fab (5GGR, 5WT9), pembrolizumab Fab (5GGS), and PD-L1 (4ZQK) were used in the structure comparison.

## Supplementary information

Supplementary Dataset 1

## Data Availability

The dataset (6K0Y) generated during the current study is available in the Protein Data Bank (https://www.rcsb.org/).

## References

[1] Chen D (2019). The FG loop of PD-1 serves as a “Hotspot” for therapeutic monoclonal antibodies in tumor immune checkpoint therapy. iScience.

[2] Park YJ, Kuen DS, Chung Y (2018). Future prospects of immune checkpoint blockade in cancer: from response prediction to overcoming resistance. Exp. Mol. Med..

[3] Couzin-Frankel J (2013). Breakthrough of the year 2013. Cancer immunotherapy. Science.

[4] Freeman GJ (2000). Engagement of the PD-1 immunoinhibitory receptor by a novel B7 family member leads to negative regulation of lymphocyte activation. J. Exp. Med..

[5] Iwai Y (2002). Involvement of PD-L1 on tumor cells in the escape from host immune system and tumor immunotherapy by PD-L1 blockade. Proc. Natl. Acad. Sci. USA.

[6] Callahan MK, Postow MA, Wolchok JD (2016). Targeting T cell co-receptors for cancer therapy. Immunity.

[7] Garon EB (2015). Pembrolizumab for the treatment of non-small-cell lung cancer. N. Engl. J. Med..

[8] Motzer RJ (2015). Nivolumab for metastatic renal cell carcinoma: results of a randomized phase II trial. J. Clin. Oncol..

[9] Topalian SL (2014). Survival, durable tumor remission, and long-term safety in patients with advanced melanoma receiving nivolumab. J. Clin. Oncol..

[10] Lee JY (2016). Structural basis of checkpoint blockade by monoclonal antibodies in cancer immunotherapy. Nat. Commun..

[11] Tan S (2017). An unexpected N-terminal loop in PD-1 dominates binding by nivolumab. Nat. Commun..

[12] Lin DY (2008). The PD-1/PD-L1 complex resembles the antigen-binding Fv domains of antibodies and T cell receptors. Proc. Natl. Acad. Sci. USA.

[13] Chen Y (2010). A dimeric structure of PD-L1: functional units or evolutionary relics?. Protein Cell.

[14] Na Z (2017). Structural basis for blocking PD-1-mediated immune suppression by therapeutic antibody pembrolizumab. Cell Res..

[15] Okada M (2017). Blockage of core fucosylation reduces cell-surface expression of PD-1 and promotes anti-tumor immune responses of T cells. Cell Rep..

[16] Yoo, S. S. *et al*. Antibodies specific to glycosylated PD-L1 and methods of use thereof. US Patent US 2018/0118830 (2017).

[17] Pinho SS, Reis CA (2015). Glycosylation in cancer: mechanisms and clinical implications. Nat. Rev. Cancer.

[18] Liu YC (2011). Sialylation and fucosylation of epidermal growth factor receptor suppress its dimerization and activation in lung cancer cells. Proc. Natl. Acad. Sci. USA.

[19] Potapenko IO (2010). Glycan gene expression signatures in normal and malignant breast tissue; possible role in diagnosis and progression. Mol. Oncol..

[20] Liang W (2018). Core fucosylation of the T cell receptor is required for T cell activation. Front. Immunol..

[21] Flocco MM, Mowbray SL (1994). Planar stacking interactions of arginine and aromatic side-chains in proteins. J. Mol. Biol..

[22] Zak KM (2015). Structure of the Complex of Human Programmed Death 1, PD-1, and Its Ligand PD-L1. Structure..

[23] Helenius A, Aebi M (2001). Intracellular functions of N-linked glycans. Science.

[24] Muro K (2015). Relationship between PD-L1 expression and clinical outcomes in patients (Pts) with advanced gastric cancer treated with the anti-PD-1 monoclonal antibody pembrolizumab (Pembro; MK-3475) in KEYNOTE-012. J. Clin. Oncol..

[25] O’Donnell PH (2015). Pembrolizumab (Pembro; MK-3475) for advanced urothelial cancer: results of a phase IB study. J. Clin. Oncol..

[26] Robert C (2015). Nivolumab in previously untreated melanoma without BRAF mutation. N. Engl. J. Med..

[27] Varki A (2017). Biological roles of glycans. Glycobiology.

[28] Scapin G (2015). Structure of full-length human anti-PD1 therapeutic IgG4 antibody pembrolizumab. Nat. Struct. Mol. Biol..

[29] Varki A (2007). Glycan-based interactions involving vertebrate sialic-acid-recognizing proteins. Nature.

[30] Fessas P, Lee H, Ikemizu S, Janowitz T (2017). A molecular and preclinical comparison of the PD-1-targeted T-cell checkpoint inhibitors nivolumab and pembrolizumab. Semin. Oncol..

[31] Tavares ABMLA, Lima Neto JX, Fulco UL, Albuquerque EL (2018). Inhibition of the checkpoint protein PD-1 by the therapeutic antibody pembrolizumab outlined by quantum chemistry. Sci. Rep..

[32] Tavares Ana Beatriz M. L. A., Lima Neto José X., Fulco Umberto L., Albuquerque Eudenilson L. (2019). A quantum biochemistry approach to investigate checkpoint inhibitor drugs for cancer. New Journal of Chemistry.

