# Supplementary material for: Study of the interactions of a novel monoclonal antibody, mAb059c, with the hPD-1 receptor
Source: Sci Rep. 2019 Nov 28;9:17830. doi: 10.1038/s41598-019-54231-w (PMC6882818; doi:10.1038/s41598-019-54231-w)
Supplement: Supplementary file 1 — Supplementary Dataset 1 [file 41598_2019_54231_MOESM1_ESM.pdf]

Study of the interactions of a novel monoclonal antibody, mAb059c, with the hPD-1 receptor

Jingxian Liu[1]*, Guiqun Wang[1], Liu Liu[1], Runjie Wu[1], Yi Wu[1], Cheng Fang[1], Xinhong Zhou[1], Jing Jiao[1], Ying Gu[1], He Zhou[1], Zhenhui Xie[1], Zhiwu Sun[1], Dakai Chen[1], Ken Dai[2], Dongxu Wang[2], Wei Tang[1], Teddy Tat Chi Yang[1,3] *

[1]Shanghai ChemPartner Co.Ltd, Shanghai, 201203, The People's Republic of China

[2]Shanghai PharmaExplorer Co., Ltd, Shanghai, 201203, The People's Republic of China

[3]Lead contact

***Corresponding authors**

Phone: 86-21-51321839; e-mail: tyang3@chempartner.com, jxliu@chempartner.com

Supplementary information

Table S1. Hydrogen bond and salt-bridge interactions.

Hydrogen Bonds (HC)

	PD1		Dist (Å)	mAb059c HC	
1	ARG 86	NE	2.7	SER 32	OG
2	ARG 86	NH1	3.2	ALA 103	O
3	ARG 86	NH2	2.9	SER 32	O
4	GLU 61	OE2	2.5	SER 57	OG

5	GLU 61	O	3.7	TYR 53	OH
6	ASP 85	OD1	2.6	SER 32	OG
7	ASP 85	OD2	2.9	SER 32	N
8	ASP 85	O	2.9	TYR 33	OH
			Salt bridge		
	ARG 86	NH1	2.8	ASP 101	OD1
	ARG 86	NH2	2.8	ASP 101	OD2

Hydrogen Bonds (LC)

	**PD1**		**Dist (Å)**	**mAb059c LC**	
1	ALA 132	N	2.8	ASP 92	O
2	PRO 130	O	3.0	TYR 94	N
			Salt bridge		
	LYS 131	NZ	2.8	ASP 92	OD1
	LYS 131	NZ	2.8	ASP 92	OD1

Table S2. Overview of identified N-glycoforms of SNTSESF and non-glycosylated SNTSESF.

Peptide	glycan mass	Glycans composition	Glycoform	XIC Area	Ratio%*

SNTSESF	NGlycan/1095.3966	HexNAc(3)Hex(3)	G0-GN	$1.64e^{+7}$	2.06
SNTSESF	NGlycan/1216.4229	HexNAc(2)Hex(5)	Man5	$5.11e^{+7}$	6.40
SNTSESF	NGlycan/1241.4545	HexNAc(3)Hex(3)Fuc(1)	G0F-GN	$2.91e^{+7}$	3.65
SNTSESF	NGlycan/1298.4760	HexNAc(4)Hex(3)	G0	$3.03e^{+7}$	3.80
SNTSESF	NGlycan/1444.5339	HexNAc(4)Hex(3)Fuc(1)	G0F	$1.38e^{+8}$	17.29
SNTSESF	NGlycan/1606.5867	HexNAc(4)Hex(4)Fuc(1)	G1F	$5.43e^{+7}$	6.80
SNTSESF	NGlycan/1647.6132	HexNAc(5)Hex(3)Fuc(1)	G0F+ GN	$4.14e^{+7}$	5.19
SNTSESF	NGlycan/1768.6395	HexNAc(4)Hex(5)Fuc(1)	G2F	$2.80e^{+7}$	3.51
SNTSESF	NGlycan/1793.6712	HexNAc(5)Hex(3)Fuc(2)	G0F2+ GN	$1.66e^{+7}$	2.08
SNTSESF	NGlycan/1809.6661	HexNAc(5)Hex(4)Fuc(1)	G1F+ GN	$3.70e^{+7}$	4.64
SNTSESF	NGlycan/2059.7349	HexNAc(4)Hex(5)Fuc(1)NeuAc(1)	G2F+NANA	$1.27e^{+8}$	15.91
SNTSESF	NGlycan/2205.7928	HexNAc(4)Hex(5)Fuc(2)NeuAc(1)	G2F2+NANA	$4.38e^{+7}$	5.49
SNTSESF	NGlycan/2336.8511	HexNAc(6)Hex(6)Fuc(1)	/	$1.80e^{+7}$	2.26
SNTS ESF	NA	NA	NA	$1.67e^{+8}$	20.93

Table S3. mAb treatment in efficacy study in MC-38 model

Group No.	Number	mAb & Dose		Date of treatment
Group 1	N=8	IgG 10mg/kg	ip x4	D0/3/6/10

Group 2	N=8	Keytruda 10mg/kg	ip x4	D0/3/6/10
Group 3	N=8	Keytruda 1mg/kg	ip x4	D0/3/6/10
Group 4	N=8	Opdivo 10mg/kg	ip x4	D0/3/6/10
Group 5	N=8	Opdivo 1mg/kg	ip x4	D0/3/6/10
Group 6	N=8	PD1-mAb059c 10mg/kg	ip x4	D0/3/6/10
Group 7	N=8	PD1-mAb059c 1mg/kg	ip x4	D0/3/6/10

Figure S1. Tumor growth curve (all groups)

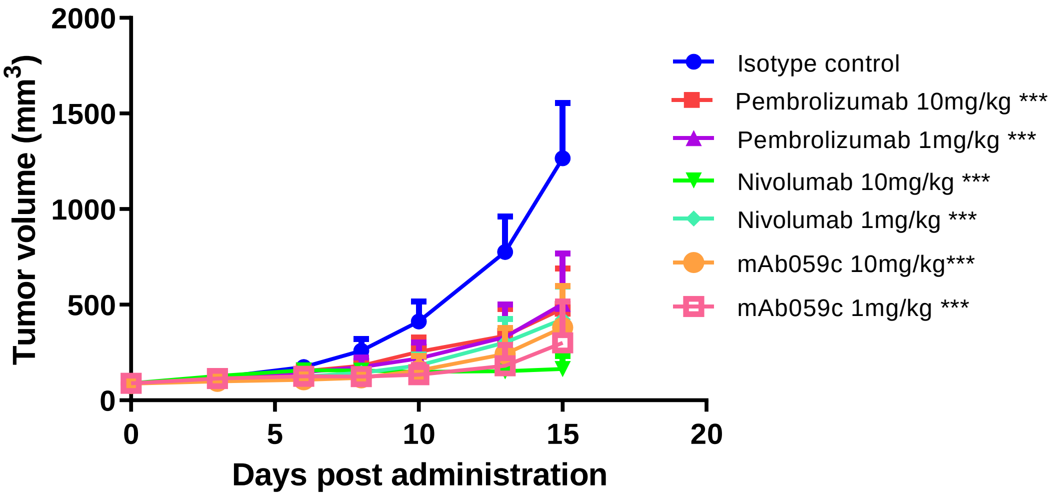

Figure S2. The full-length gel image as shown in Figure 1d.

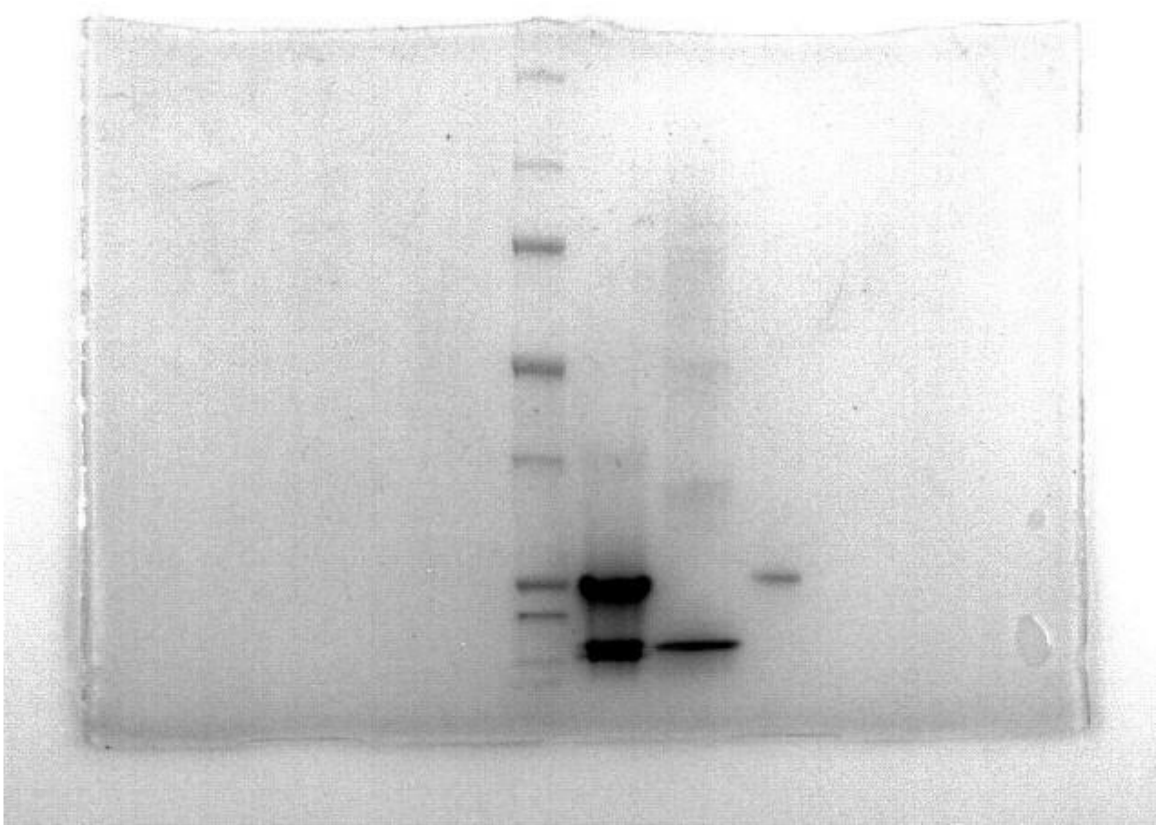

Supplementary Methods

Generation of fully human Anti-PD-1 antibody mAb059c

Generation of fully human anti-human PD-1 antibody mAb059c has been reported and published (PCT/CN2016/091962; Jul 27, 2016). Briefly, Anti-human PD-1 antibody mAb059c was generated with hybridoma approach by immunizing 6-8 week old Harbour H2L2 fully human transgenic mice with recombinant human PD-1 extracellular domain Fc fusion protein. Hybridoma clones with specific binding to human and cynomlgous PD-1, blocks PD-1 and PD-L1 interaction were selected for subcloning and hybridoma antibodies were purified. Purified Hybridoma antibodies were characterized by their binding to CHOK1 cells stably expressing human PD-1, cross reactivity to cynomlgous and mouse PD-1, selectivity to PD-1 but not to other B7 family members by protein based ELISA assay. The effect of the purified antibodies on blocking PD-1 and PD-L1 interaction as well as activation of T cell functions in T cell stimulation assay, mixed lymphocytes reactions were investigated. Among all the purified hybridoma antibodies, mAb059c showed the highest affinity and potency in various cell based functional assays such as mixed lymphocytes reaction. Heavy chain and light chain variable regions of mAb059c were sequenced and reformatted into human IgG4 backbone to generate fully human anti-PD-1 mAb059c antibody.

LIU, Lile; YANG, Xinxiu; LUO, Haishan; LIU, Hu; SHUAI, Zhengrong; WANG, Jian; ZHONG, Qin; DUAN, Qing; GU, Hongzhuan; YANG, Tatchi Teddy, inventors; PharmaExplorer, assignee; Anti-PD-1 antibodies and uses thereof. PCT/CN2016/091962; Jul 27, 2016

Glycan analysis

100 µg sample was added into 200 µl denature solution (8 M Guanidine HCl, pH 6.5) and mixed well. 4 µl 1M DTT was added into the tube to the final concentration of 20mM. The mixture was incubated at 37 °C for 1 h to reduce all the disulfide bonds. The alkylation was performed by adding 8 µl 1M IAM into the solution in the dark and the reaction lasted for 30 min.

Before trypsin digestion, the sample was buffer exchanged into 50mM TEAB by ultrafiltration. 300 µl 50mM TEAB (pH 6.5) was added into the centrifugal filter and the filter was centrifuged at 10,000 rpm for 5 min. The sample was transferred to the centrifugal filter and centrifuged at 10,000 rpm for 15 min. This step was repeated three times. Invert each filter cartridge into a new labeled collection tube and centrifuge at 2,000 rpm for 5 min. More 50mM TEAB (pH6.5) was added into the solution until the final

volume 100 µl was reached. 2 µg chymotrypsin was added into the sample and Incubated at 37 ℃ for 2 h. 5 µl 10% FA was added into the tube to stop the digestion.

After chymotrypsin digestion, the N58N-glycan site was located in the peptide of SNTSESF. 13 different N-glycoforms and deglycosylated peptide were detected by HPLC-MS and database search. All the tandem mass spectra of glycopeptides were checked manually to ensure the oxonium ions were generated by higher-energy collisional dissociation. Details of glycopeptides including glycan types and relative abundance were summarized in the Table S2.
